# On offer to Ontario consumers three years after legalization: A profile of cannabis products, cannabinoid content, plant type, and prices

**DOI:** 10.3389/fpsyt.2023.1111330

**Published:** 2023-02-16

**Authors:** Felicia Tassone, Patricia Di Ciano, Yuxin Liu, Sergio Rueda

**Affiliations:** ^1^Institute for Mental Health Policy Research, Centre for Addiction and Mental Health, Toronto, ON, Canada; ^2^Campbell Family Mental Health Research Institute, Centre for Addiction and Mental Health, Toronto, ON, Canada; ^3^Department of Pharmacology and Toxicology, Temerty Faculty of Medicine, University of Toronto, Toronto, ON, Canada; ^4^Department of Psychiatry, Temerty Faculty of Medicine, University of Toronto, Toronto, ON, Canada; ^5^Institute of Health Policy, Management and Evaluation, University of Toronto, Toronto, ON, Canada

**Keywords:** cannabis legalization, legal market, adult consumers, cannabis products, cannabis prices, cannabis potency, THC, CBD

## Abstract

**Introduction:**

Cannabis was legalized in Canada in October 2018, regulating the production, distribution, sale, and possession of dried cannabis and cannabis oils. Additional products were legalized 1 year later, including edibles, concentrates, and topicals, with new lines of commercial products coming to market. Ontario is the most populous province in Canada and has the largest cannabis market with the highest number of in-person retail stores and the most cannabis products available online. This study aims to create a profile of products available to consumers three years after legalization by summarizing types of products, THC and CBD potency, plant type, and prices of product sub-categories.

**Methods:**

We extracted data from the website of the Ontario Cannabis Store (OCS)—the public agency overseeing the only online store and sole wholesaler to all authorized in-person stores—in the first quarter of 2022 (January 19–March 23). We used descriptive analyses to summarize the data. A total of 1,771 available products were mapped by route of administration into inhalation (smoking, vaping, and concentrates), ingestible (edibles, beverages, oils, and capsules) and topical.

**Results:**

Most inhalation products included ≥20%/g THC (dried flower: 94%; cartridges: 96%; resin: 100%) while ingestible products had similar proportions of THC and CBD content. Indica-dominant products tend to be more prominent in inhalation products while sativa-dominant products tend to be more prominent in ingestible products. The average sale price of cannabis was 9.30 $/g for dried flower, 5.79 $/0.1g for cartridges, 54.82 $/g for resin, 3.21 $/unit for soft chews, 1.37 $/ml for drops, 1.52 $/unit for capsules, and 39.94 $/product for topicals.

**Discussion:**

In summary, a wide variety of cannabis products were available to Ontarians for different routes of administration and provides numerous indica-dominant, sativa-dominant, and hybrid/blend options. The current market for inhalation products however is geared towards the commercialization of high-THC products.

## Introduction

Canada was the second nation globally to legally regulate the production, distribution, sale, and possession of dried cannabis and cannabis oils in October of 2018 (1). A “second wave” of legalization came into effect in October 2019, regulating edibles, high-potency concentrates, and topicals (2), with new lines of commercial products available by early 2020. One of the main goals of legalization was to provide safe, legal access to cannabis products (3), enabling the legal industry to compete with the illegal market. The legalization of non-medical cannabis in Canada occurred under the Cannabis Act, which regulates cannabis nationally, but under Canada’s constitutional division of powers, each of the 10 provinces and 3 territories developed their own laws and regulatory systems (1). Ontario, Canada’s most populous province and largest cannabis market, follows a hybrid model where a public agency is the sole wholesaler to all authorized in-person stores—all of which are private—and also provides direct access to the public through the only online store.

Purchasing behaviors of consumers are influenced by cannabis prices and availability in the illegal and legal markets (4). In the US, cannabis use is higher in states that have legalized cannabis, with dried cannabis being the most dominant form (5). It is too early to determine the impact of legalization in Canada as the legal market continues to evolve, but early evidence suggests increased use in adults, mixed effects in adolescent use and on driving under the influence of cannabis, increases in pediatric emergency room visits and hospitalizations, and decreases in arrests and convictions (6–13). Given the acute and long-term health risks associated with cannabis containing high levels of THC (14–17), documenting the potency of the cannabis products available in the legal market is of interest to public health.

One year post-legalization, a Canadian study found that the top 10% of cannabis users (those with the highest cumulative cannabis use) accounted for about two-thirds of cannabis consumption, with 40% of the cannabis consumed in the form of flower products (18). At that time, legal cannabis sales covered about 33% of Canada’s cannabis consumption (19). Two years post-legalization, there were a total of 1,183 legal cannabis stores in Canada (20). Three years post-legalization, Ontario had the highest number of in-person retail stores (*n* = 1,974) and the highest number of products available online (*n* = 1,685) among all the provinces and territories (21, 22). The legal market has expanded to an estimated 57% of sales as of the last quarter of 2021, making progressive gains in the displacement of the illegal market (23).

Our aim was to create a profile of the products available to adult consumers in Ontario—just over three years post-legalization—and report their THC and CBD potency, plant type (e.g., indica-dominant, sativa-dominant, hybrid), and price. To do so, we cataloged products listed on the website of the Ontario Cannabis Store (OCS), the public agency overseeing the only online store and sole wholesaler to all authorized in-person stores. We do not present sales data which are available elsewhere for approximately the same period (23).

## Materials and methods

This is a cross-sectional study designed to produce a profile of the legal cannabis market in Ontario three years post-legalization. We extracted data from the OCS website to document the THC and CBD potency, plant type, and price of all products available to purchase by Ontario consumers over a span of two months (19 January–23 March 2022) (22). During data extraction, FT and YL manually entered information from each cannabis product into an Excel spreadsheet. Any unavailable or unlisted information was marked as “N/A”. We used descriptive statistics [counts, means (*M*), standard deviations (SD), and ranges] using Excel functions. FT used pivot tables, sorted columns alphabetically, and counted each product individually to determine counts of high potency products and plant types. We also mapped the product categories and subcategories provided in the OCS website by route of administration to provide a more meaningful consumer perspective.

Products on the OCS website are listed with a range of values for both THC and CBD content (in%/g or mg/unit), rather than a single THC or CBD value as it is presented on the label of the individual product purchased by consumers. By design, regulations placed THC limits per package on ingestible products (e.g., maximum of 10 mg of THC per package for edibles). Thus, we calculated the average for each product THC and CBD range and then averaged the THC and CBD potencies of all products by their sub-category. Also, the OCS labels products that contain 20%/g or greater THC as “very strong THC” levels (OCS (22)). We calculated the frequency and proportion of very strong THC products using the unit%/g from each product. As the OCS does not define “very strong CBD” levels, we labeled any product with an average value above 5%/g as very strong CBD. We then calculated the number of very strong THC and CBD products by product sub-category using pivot tables, alphabetical sorting, and manual counting. For plant type, we sorted alphabetically and manually counted all the products categorized as blend, hybrid, indica-dominant, or sativa-dominant for each of the sub-categories.

In terms of price, a single product often had multiple selling prices listed depending on the quantity available for purchase, so we calculated the average of the lowest and highest selling price for each product and then calculated the average price for each sub-category (in $/g, $/0.1g, $/ml, or $/unit). In addition, we calculated the minimum selling price and the maximum selling price for each sub-category. These average selling prices allow us to approximate an estimate of how much money a consumer would spend to purchase a particular product in bulk.

Our study cataloged all the products available in the OCS during the period of observation and most dried flower products were only available in one or two quantities while pre-roll products were available in 20 different quantities (ranging from 0.25 to 30g), making it difficult to report average prices for pre-determined quantities as previous studies have done (24–27). Thus, the lowest and highest price per gram of every product was used for clarity.

The top 10 cannabis products by units sold in Ontario retail stores were listed in the OCS’s January to March 2022 quarterly review (23). We used our Excel spreadsheet with the data extracted from the OCS website to provide more information about each of these ten products, including THC and CBD potency, plant type, and price.

## Results

### Product types and routes of administration

We mapped a total of 1,771 available products by route of administration into inhalation (smoking, vaping, and concentrates: *n* = 1,250), ingestible (edibles, beverages, oils, and capsules: *n* = 410), topical (*n* = 75), and other (pantry, seeds, and starter kits: *n* = 36) (Tables 1, 2). The sub-categories with the most products under each of the nine categories were: dried flower (*n* = 508), thread cartridges (*n* = 230), resin (*n* = 50), soft chews (*n* = 104), beverages (*n* = 106), oils-drops (*n* = 80), capsules (*n* = 39), and topicals (*n* = 75) (Table 2).

**TABLE 1:** Ontario cannabis store (OCS) product categories and sub-categories mapped by route of administration (RoA).

OCS categories	OCS sub-categories	RoA categories	RoA sub-categories
Flower	Dried flower	Inhalation—smoking	Dried flower
	Pre-rolls		Pre-rolls
	Seeds	Inhalation—vaping	510 thread cartridges
Vapes	510 thread cartridges		Pax pods
	Pax pods		Closed loop pods
	Closed loop pods		Disposable vape pens
	Disposable vape pens	Inhalation—concentrates	Hash
	Starter Kits		Kief and sift
Extracts	Oils		Resin
	Capsules		Rosin
	Hash		Shatter and wax
	Kief and sift		Isolates and distillates
	Resin	Ingestible—edibles	Soft chews
	Rosin		Chocolates
	Shatter and wax		Baked goods
	Isolates and distillates		Hard edibles
Edibles	Soft chews	Ingestible—beverages	Beverages
	Chocolates	Ingestible—oils	Drops
	Beverages		Spray
	Baked goods	Ingestible—capsules	Capsules
	Hard edibles	Topicals	Topicals
	Pantry	Others	Pantry
CBD and topicals	Topicals		Seeds
			Starter kits

**TABLE 2:** Tetrahydrocannabinol (THC) and cannabidiol (CBD) potency by product type (first quarter of 2022).

Category	No. of products	*M (SD)* [Range]		No. of very strong products (%)	
		THC	CBD	THC (≥ 20%/g)	CBD (≥ 5%/g)
**Inhalation—smoking (%/g)**					
Dried flower	508	*22 (4)* [0–33]	*1 (2)* [0–20]	476 (94)	30 (6)
Pre-rolls	305	*21 (4)* [0–36]	*1 (2)* [0–20]	283 (93)	15 (5)
**Inhalation—vaping (%/g)**					
Thread cartridges	230	*74 (19)* [0–96]	*6 (7)* [0–90]	221 (96)	44 (19)
Pax pods	21	*74 (12)* [39–88]	*5 (12)* [0–46]	21 (100)	6 (29)
Closed loop pods	3	*59 (42)* [11–89]	*20 (34)* [0–61]	2 (67)	1 (33)
Disposable pens	16	*65 (29)* [11–92]	*18 (27)* [0–71]	14 (88)	5 (31)
**Inhalation—concentrates (%/g)**					
Hash	31	*44 (14)* [17–71]	*2 (6)* [0–34]	31 (100)	3 (10)
Kief and sift	17	*34 (4)* [25–47]	*1 (1)* [0–10]	17 (100)	1 (6)
Resin	50	*71 (16)* [15–96]	*4 (11)* [0–64]	50 (100)	11 (22)
Rosin	24	*68 (12)* [27–85]	*2 (2)* [0–10]	24 (100)	6 (25)
Shatter and wax	31	*75 (3)* [63–85]	*2 (2)* [0–10]	31 (100)	10 (32)
Isolates and Distillates	14	*46 (36)* [0–96]	*33 (39)* [0–102]	9 (64)	8 (57)
**Ingestible—edibles (mg/unit, mg)**					
Soft chews	104	*4 (3)* [0–10]	*4 (6)* [0–25]	N/A	N/A
Chocolates	46	*8 (3)* [0–10]	*3 (6)* [0–25]	N/A	N/A
Baked goods	16	*7 (3)* [2–10]	*1 (2)* [0–5]	N/A	N/A
Hard edibles	12	*2 (1)* [1–10]	*4 (8)* [0–20]	N/A	N/A
**Ingestible—beverages (mg/unit, mg)**					
Beverages	106	*4 (4)* [0–10]	*6 (8)* [0–45]	N/A	N/A
**Ingestible—oils (%/g, mg/spray)**					
Oils—drops	80	*1 (11)* [0–4]	*2 (25)* [0–11]	0 (0)	8 (10)
Oils—spray	7	*2 (1)* [0–5]	*2 (4)* [0–13]	0 (0)	N/A
**Ingestible—capsules (mg/unit, mg)**					
Capsules	39	*4 (4)* [0–10]	*10 (14)* [0–53]	N/A	N/A
**Topicals (mg)**					
CBD and topicals	75	*69 (116)* [0–500]	*280 (259)* [5–1500]	N/A	N/A
**Other**					
Pantry (mg)	4	*8 (5)* [0–10]	*250 (500)* [0–1000]	N/A	N/A
Seeds	30	N/A	N/A	N/A	N/A
Starter kits (%/g)	2	*42 (54)* [2–88]	*40 (56)* [0–84]	1 (50)	1 (50)

### THC and CBD potency

A large proportion of inhalation products were categorized as very strong THC products (percent of products with ≥20%/g THC and average THC): dried flower: 94%, 22%/g; thread cartridges: 96%, 74%/g; resin: 100%, 71%/g (Table 2). The trend of high-THC products continued across the inhalation sub-categories, with all sub-categories having over 64% of products being classified as having very strong THC levels and 100% for most concentrates and some vaping products. Apart from isolates and distillates and closed loop pods, all sub-categories had very strong levels of THC in over 88% of products. On the other hand, only 6% of dried flower and 5% of pre-rolls had CBD levels ≥5%/g. Except for isolates & distillates, all sub-categories had lower than 33% of products with very strong CBD levels. Topical products averaged 69 mg/product with a range of 0–500 mg.

No ingestible products exceeded the regulatory limit of THC per package (28). As such, ingestible products saw lower average THC values than inhalation products, including soft chews, beverages, and capsules at 4 mg/unit, and drops at 2%/g (Table 2). The average CBD content in ingestible products corresponded more closely to the THC levels within those products: soft chews: 4 mg/unit; beverages: 6 mg/unit; drops: 2%/g; capsules: 10 mg/unit; and topicals: 280 mg/product.

### Plant type

The vast majority of products provided information on the plant type (i.e., blend, hybrid, indica-dominant, sativa-dominant) (Table 3); this information was missing for only 10 products (0.56%).

**TABLE 3:** Plant type by product type (first quarter of 2022).

Category	No. of products				
	Total	Blend	Hybrid	Indica-dominant	Sativa-dominant
**Inhalation—smoking**					
Dried flower	508	4 (1%)	125 (25%)	244 (48%)	133 (26%)
Pre-rolls	305	8 (3%)	87 (29%)	131 (43%)	77 (25%)
**Inhalation—vaping**					
Thread cartridges	230	36 (16%)	51 (22%)	79 (34%)	64 (28%)
Pax pods	21	3 (14%)	3 (14%)	6 (29%)	9 (43%)
Closed loop pods	3	0 (0%)	1 (33%)	2 (67%)	0 (0%)
Disposable pens	16	1 (6%)	6 (6%)	4 (25%)	5 (31%)
**Inhalation—concentrates**					
Hash	31	8 (26%)	11 (35%)	8 (26%)	4 (13%)
Kief and sift	17	1 (6%)	5 (29%)	6 (35%)	5 (29%)
Resin	50	1 (2%)	17 (34%)	17 (34%)	15 (30%)
Rosin	24	0 (0%)	8 (33%)	13 (54%)	3 (13%)
Shatter and wax	31	1 (3%)	15 (48%)	10 (32%)	5 (16%)
Isolates and distillates	14	3 (21%)	5 (36%)	0 (0%)	5 (36%)
**Ingestible—edibles**					
Soft chews	104	44 (42%)	34 (33%)	11 (11%)	15 (14%)
Chocolates	46	27 (59%)	17 (37%)	1 (2%)	1 (2%)
Baked goods	16	11 (69%)	1 (6%)	1 (6%)	3 (19%)
Hard edibles	12	11 (92%)	1 (8%)	0 (0%)	0 (0%)
**Ingestible—beverages**					
Beverages	106	60 (57%)	18 (17%)	2 (2%)	26 (25%)
**IIngestible—oils**					
Oils—drops	80	50 (63%)	13 (16%)	6 (8%)	8 (10%)
Oils—spray	7	3 (43%)	1 (14%)	1 (14%)	2 (29%)
**Ingestible—capsules**					
Capsules	39	12 (31%)	12 (31%)	4 (10%)	10 (26%)
**Topicals**					
CBD and topicals	75	30 (40%)	27 (36%)	5 (7%)	13 (17%)
**Other**					
Pantry	4	4 (100%)	0 (0%)	0 (0%)	0 (0%)
Seeds	30	0 (0%)	22 (73%)	2 (7%)	4 (13%)
Starter kits	2	2 (100%)	0 (0%)	0 (0%)	0 (0%)

Relative to ingestible products, inhalation products tended to have a higher percentage of indica-dominant than sativa-dominant products (dried flower: 48% vs. 26%, cartridges: 34% vs. 28%, resin: 34% vs. 30%) while the opposite was true for ingestible products (soft chews: 11% vs. 14%, beverages: 2% vs. 25%, drops: 8% vs. 10%, capsules: 10% vs. 26%, and topicals: 7% vs. 17%).

Ingestible products had more hybrid and blend products available relative to inhalation products, with the majority of the ingestible sub-categories carrying mainly blend or hybrid products. All edibles sub-categories classified over 75% of products as either hybrid or blend, with less than 25% being classified as indica- or sativa-dominant.

### Price

Typically, products were available at a lower selling price when purchasing higher amounts (Table 4). This enables consumers to buy more cannabis for a lower price per gram. For example, if a product’s lowest selling price was $39.95 for 4 g and its highest selling price was $129.95 for 14 g, the lowest price in $/g would be $9.99 for 4 g and $9.28 for 14 g. The consumer has the option to save $0.71/g by buying more cannabis. This was true in the vast majority of cases, but not in all cases.

**TABLE 4:** Average prices by product type (first quarter of 2022).

Category	Sub-category	*M (SD)* [range]	
		Price	Selling price
Inhalation—smoking		$/g	$
	Dried flower	*9.30 (3.33)* [3.57–17.13]	*51.39 (35.66)* [4.50–259.95]
	Pre-rolls	*11.85 (4.01)* [4.82–24.87]	*21.99 (12.65)* [4.88–135.95]
Inhalation—vaping		$/0.1g	$
	Thread cartridges	*5.79 (1.82)* [3.50–17.99]	*40.99 (10.72)* [15.95–89.95]
	Pax pods	*8.94 (1.88)* [5.79–11.99]	*43.43 (10.03)* [28.95–59.95]
	Closed loop pods	*9.66 (0.58)* [8.99–9.99]	*48.28 (2.89)* [44.95–49.95]
	Disposable pens	*12.06 (4.57)* [6.65–17.98]	*36.64 (10.96)* [16.95–44.95]
Inhalation—Concentrates		$/g	$
	Hash	*28.73 (13.24)* [9.99–49.95]	*36.39 (9.66)* [14.95–49.95]
	Kief and sift	*17.18 (3.59)* [10.47–22.95]	*24.01 (8.63)* [14.95–39.96]
	Resin	*54.82 (16.31)* [13.97–109.90]	*53.43 (16.22)* [20.96–124.95]
	Rosin	*62.92 (19.65)* [16.63–109.95]	*57.78 (20.08)* [24.95–109.95]
	Shatter and wax	*48.66 (13.37)* [26.48–79.90]	*44.50 (16.14)* [17.48–79.90]
	Isolates and distillates	*45.85 (8.62)* [32.95–65.90]	*37.23 (10.52)* [13.80–44.95]
Ingestible—edibles		$/unit	$/package
	Soft chews	*3.21 (1.92)* [0.43–8.95]	*10.00 (7.72)* [3.98–44.95]
	Chocolates	*4.34 (1.45)* [1.39–7.45]	*5.50 (1.49)* [2.48–10.20]
	Baked goods	*5.09 (2.33)* [1.65–9.75]	*7.50 (2.15)* [2.95–9.95]
	Hard edibles	*1.82 (0.67)* [1.00–3.33]	*10.08 (6.02)* [5.95–24.95]
Ingestible—beverages		$/unit	$/package
	Beverages	*6.11 (1.94)* [2.40–13.80]	*8.14 (4.47)* [2.95 –21.95]
Ingestible—oils		$/ml	$
	Oils—drops	*1.37 (0.60)* [0.47–3.60]	*37.20 (14.78)* [13.95–89.95]
	Oils—spray	*1.47 (0.36)* [1.20–2.00]	*25.66 (3.55)* [19.95–29.95]
Ingestible—capsules		$/unit	$/package
	Capsules	*1.52 (1.18)* [0.63–7.75]	*26.79 (13.05)* [9.50–74.95]
Topicals			$
	CBD and topicals	N/A	*39.94 (15.53)* [8.95–82.20]
Other		$/0.1g	$
	Pantry	N/A	*19 (22.33)* [3.95–51.95]
	Seeds	N/A	*46.59 (9.64)* [24.95–64.95]
	Starter kits	*8.29 (1.88)* [6.59–9.99]	*41.45 (10.03)* [32.95–49.95]

The average selling price of cannabis products was $51.39 (4.50–259.95) for dried flower, $40.99 (15.95–89.95) for cartridges, $53.43 (20.96–124.95) for resin, $10.00/package (3.98–44.95) for soft chews, $37.20 (13.95–89.95) for drops, $26.79/package (9.50–74.95) for capsules, and $39.94 (8.95–82.20) for topicals.

The average price per unit of cannabis for the most numerous sub-categories was found to be 9.30 $/g (3.57–17.13) for dried flower, 5.79 $/0.1g (3.50–17.99) for cartridges, 54.82 $/g (13.97–109.90) for resin, 3.21 $/unit (0.43–8.95) for soft chews, 1.37 $/ml (0.47–3.60) for drops, and 1.52 $/unit (0.63–7.75) for capsules.

Many products in the flower and pre-roll sub-categories were sold at multiple price points due to the variety of quantities available for purchase. A product in the flower section could be available in multiple sizes, including 1, 3.5, 5, 7, 10, 11, 14, 15, 28, or 30 g. Similarly, products in the pre-roll section could be available in 0.25, 0.3, 0.32, 0.33, 0.35, 0.4, 0.42, 0.5, 0.6, 0.7, or 1 g. For each of these sizes, a different number of pre-rolls may be available, ranging from 1 to 70. Thus, one product could have multiple price points due to being available in a variety of quantities.

### Top 10 cannabis products by units sold

In the first quarter of 2022, the top ten cannabis products by units sold in Ontario retail stores were mainly inhalation products (Table 5). Eight of the 10 products fall under the dried flower (*n* = 5) and pre-roll (*n* = 3) sub-categories. Two of the top 10 products sold were ingestible and fall under the soft chews sub-category. All inhalation products had “very strong” THC levels with an average of 21%/g. Inhalation products had an average price of 6.49 $/g while ingestible products had an average price of 2.77 $/unit.

**TABLE 5:** Characteristics of top 10 cannabis products by units sold in Ontario retail stores (first quarter of 2022).

Rank	RoA Sub-Category	Brand	Product name	Average THC	Average CBD	Potency	Plant type	Price	Selling price
1	Dried Flower	SHRED	Tropic thunder	21%/g	0.50%/g	Very Strong	Hybrid	4.56 $/g	$31.95/7g
2	Dried flower	SHRED	Funk master	21%/g	0.75%/g	Very Strong	Hybrid	4.56 $/g	$31.92/7g
3	Pre-rolls	Good supply	Jean guy pre-roll	21%/g	1.00%/g	Very Strong	Sativa dominant	8.65 $/g	$8.65/1g
4	Pre-rolls	Good supply	Grower’s choice Indica pre-roll	21%/g	0.50%/g	Very Strong	Indica dominant	6.50 $/g	$6.50/1g
5	Dried flower	Spinach	GMO cookies	23%/g	0.08%/g	Very Strong	Indica dominant	9.99 $/g	$34.97/3.5g
6	Dried flower	Pure sunfarms	Pink kush	23%/g	0.50%/g	Very Strong	Indica dominant	6.56 $/g	$22.95/3.5g
7	Dried flower	SHRED	Gnarberry	20%/g	0.50%/g	Very Strong	Hybrid	4.56 $/g	$31.92/7g
8	Soft chews	Spinach	SOURZ by spinach - blue raspberry watermelon Indica	10 mg	0.10 mg	N/A	Indica dominant	1.56 $/unit	$7.80/1 pack
8	Soft chews	Wana	Pomegranate blueberry Acai 5:1 sour soft chews	10 mg	50.00 mg	N/A	Blend	3.98 $/unit	$7.95/1 pack
10	Pre-rolls	Good supply	Grower’s choice Sativa pre-roll	19%/g	0.50%/g	Very strong	Sativa dominant	6.50 $/g	$6.50/1g

## Discussion

This study describes the Ontario cannabis legal market three years after legalization by cataloging every cannabis product on offer to consumers rather than profiling a market based on a subset or a random selection of products. There was a large variety of products on offer to Ontario consumers with inhalation products being 2.5 times more numerous than ingestible products. The majority of inhalation products had very strong levels of THC. They also had a higher percentage of indica- than sativa-dominant products while ingestible products saw the opposite trend. The average price per unit of dried cannabis was $9.30/g, which is lower than self-reported data collected prior to legalization (29, 30).

Product variety included varying routes of administration, with 46% of items classified as smoking products, 16% as vaping, ∼10% each for edibles and concentrates, and between 2 and 6% each for beverages, oils, capsules, and topicals. The breakdown of product types resembled that for total sales during the same time period, which was 50% for dried flower, 18% pre-rolls, 16% vapes, 5% each of edibles and concentrates, and 2% each of oils and beverages (23). We also found that eight out of the top ten products sold in retail stores were smoking products. In the 3 years post-legalization, consumer preferences for particular types of cannabis products have shifted, although smoking continues to be the most common route of administration (31). From 2017 to 2022, the Canadian Cannabis Survey—a national survey implemented by the Government of Canada to monitor the effects of legalization—reported a decrease in smoking (94–70%) and vaping using vaporizers (14–10%), and an increase in vaping using vape pens (20–31%) and edibles (34–52%) (31, 32). From 2020 to 2022, of those who vaped cannabis, liquid cannabis oil/concentrate use increased (60–74%) and dried flower use decreased (65–49%) (31, 33). This shift in consumer preferences is generally seen as a positive consequence of legalization as lower-risk cannabis use guidelines recommend avoiding routes of administration that involve smoking combusted cannabis products (15). However, the guidelines also caution consumers about the risk of ingesting larger than anticipated doses associated with edibles, which have been linked with increased emergency room visits and hospitalizations, especially with pediatric populations (9, 10).

We observed that the current legal cannabis market for inhalation products is geared toward offering products with high THC potency with a lower availability of balanced THC-CBD or high-CBD options. The average THC potency in the current market is higher than the average potency of 10-13% reported by Health Canada prior to legalization (29), and higher still than the average 6–10% reported in the legal medical market that existed pre-legalization of non-medical cannabis (30). THC potency had already been increasing prior to legalization with a 212% increase from 1995 to 2015 (34). At the time of legalization in late 2018, the average THC potency of legal dried flower across a sample of legal retail stores in Ontario was 16% (31). As of early 2022, we observed that THC levels in dried cannabis on offer to Ontario consumers has an average of 22%. Consumers should be aware that high potency cannabis can have detrimental effects on mental health and addiction outcomes (16, 17, 35–37). Within this context, some have argued for imposing limits on THC in cannabis products, and others propose the use of excise tax based on THC levels to incentivize the use of less potent products (38). It is unknown to what degree THC levels impact consumer product choice; however, in various studies of consumer views on cannabis quality, the potency of cannabis was not mentioned as a marker (31, 39–41). In the latest Canadian Cannabis Survey, strength was ranked last among the factors that influence consumer purchases after price, safe supply, quality, convenience, proximity to retailer, and ability to purchase from a legal source (31). Public outreach around the safer use of cannabis should consider lower-risk cannabis use guidelines; one of the recommendations of which is the choice of lower-potency cannabis products (15). Given the availability of more potent cannabis, future experimental research into their health effects is needed as there is still uncertainty over whether consumers effectively self-titrate THC doses of higher potency products (42).

Despite strict regulations on testing requirements for cannabinoids and contaminants on cannabis products in the Canadian market, we are not aware of peer-reviewed, independent evaluations of the accuracy of product labels (43, 44). However, US studies have reported inconsistencies in cannabinoid labeling on commercial products (45–47). In addition, it has been shown that consumers have difficulties understanding and applying quantitative cannabinoid labeling and additional work should be devoted to improve the labeling of standard doses across routes of administration (48, 49).

The legal market offered a wide variety of products containing different plant types. Inhalation products had higher percentages of indica-dominant products relative to all other plant types, while ingestible products had higher percentages of sativa-dominant products. There were many offers of blend or hybrid options available in every product category. The taxonomical classification of cannabis into indica and sativa are mostly based on marketing considerations as they do not exist in nature due to historical cross-breeding and misuse of nomenclature (50, 51). However, cannabis users report differential subjective experiences between indica- and sativa-dominant products with greater preference for using indica in the evening while reporting feeling “relaxed, sleepy/tired” and sativa during the day while reporting feeling “alert/energized” (52). Overall, offered products appear consistent with consumer preferences for a variety of indica-dominant, sativa-dominant, and hybrid/blend products.

Previous studies of cannabis pricing have typically been done by survey research (24, 25), by selecting a subset of products (e.g., most popular, least, and most expensive) (26), or by calculating the average price of a random sample of products at each pre-determined purchase quantity (27). These studies have typically reported on the average price of dried flower and pre-rolls at set quantities (e.g., the average price at 1, 3.5, 7, 14, and 28 g). In our study, the average price per gram of dried flower for all products on offer was found to be $9.30 and the lowest price was $3.41 per gram. Sales during that period suggest that the majority of consumer purchases were made in the lower price range, with most of the purchases between $3.00 and 6.50 per gram making up 48% of in-person purchases and 63% of online purchases (23). Our calculations suggest that spending more money on larger quantities would provide consumers with a “better deal,” with the average selling price of dried flower products being $51.39. In line with 2022 self-reported data, consumers who had used recreational cannabis within the last 30 days spent an average of $65 ($46–$86) from legal sources per month, an increase from $55 in 2021 (31, 39). Participants who used cannabis for medical purposes spent an average of $75 in the past 30 days (31). For consumers that tend to spend above $50 a month on cannabis, buying dried flower products “in bulk” seems to be a reasonable approach for reducing the cost of cannabis per gram.

The average dried flower prices was lower than those reported in studies of the retail cannabis market in Canada pre-legalization and at the time of legalization (29, 30). Prior to legalization, the average self-reported price-per-gram of cannabis was $9.56, but this varied depending on the quantity purchased (24). In comparison to self-reported data collected several months post-legalization, the average price of legal dried flower has decreased slightly: 9.82 $/g in late 2018 versus 9.30 $/g in early 2022 (27). In comparison to self-reported legal cannabis prices in Canada from 2019, regardless of quantity, the average price of dried flower in 2022 is also lower, ranging from $23.16/1g to $9.95/3.5 g and $9.95/27.9 g in 2019 (25). Additionally, it was found that the price of dried flower from legal sources decreased post-legalization (25), consistent with our findings.

In a recent qualitative study from Canada, participants spoke of the benefits associated with the illegal cannabis market, including lower prices, incentives, discounts, and loyalties (41). The majority of participants in that study noted that legal cannabis is more expensive than illegal cannabis and that price was of highest importance when making purchasing decisions (41). This is in agreement with the Canadian Cannabis Survey in which 30% of participants in 2022 ranked price as the number one factor that influences purchases (31, 39). Users on social media platforms thought legal products were expensive in comparison to illegal cannabis and have also expressed concerns with difficulties with supply shortages in the legal market (53). Cannabis users have indicated that price, product quality, store location, and the inconvenience of purchasing from legal sources were key reasons for buying cannabis from the illicit market (54). However, if the average price per gram and average selling prices of the legal market continue to follow downward trends, then more Canadians may become motivated to purchase from legal sources (54). Recent Canadian research states that the divergence between legal and illegal cannabis markets is narrowing (25) and the legal share of the overall cannabis market is estimated to be 57% (23).

## Conclusion

The Ontario legal market has a wide variety of cannabis products on offer to consumers. There is a wide array of products available for various consumer preferences in plant type, with numerous indica-dominant, sativa-dominant, and hybrid/blend options on offer. In line with lower-risk cannabis use guidelines (15), consumers would benefit from having access to more lower-potency, mixed THC-CBD ratios, and CBD-dominant products. Three years post-legalization, Canadian cannabis consumers have favorable perceptions of legal cannabis products in comparison to illegal products, apart from price (40).

## Data availability statement

The raw data supporting the conclusions of this article will be made available by the authors, without undue reservation.

## Author contributions

SR and PD conceived, designed, and directed the study. FT extracted the data with support from YL. FT analyzed the data and wrote the first draft of the manuscript. SR carried out substantial edits to the initial draft. All authors reviewed various drafts and approved the final version of the manuscript.
